# Aerobic bacteria in post surgical wound infections and pattern of their antimicrobial susceptibility in Ayder Teaching and Referral Hospital, Mekelle, Ethiopia

**DOI:** 10.1186/1756-0500-7-575

**Published:** 2014-08-27

**Authors:** Reiye Esayas Mengesha, Berhe Gebre-Slassie Kasa, Muthupandian Saravanan, Derbew Fikadu Berhe, Araya Gebreyesus Wasihun

**Affiliations:** Department of Surgery Mekelle University, College of Health Sciences, P.O. Box 1871, Mekelle, Ethiopia; Department of Medical Microbiology, Mekelle University College of Health Sciences, P.O. Box 1871, Mekelle, Ethiopia; Department of Pharmacy Mekelle University, College of Health Sciences, P.O. Box 1871, Mekelle, Ethiopia

**Keywords:** Surgical ward, Post-surgical wound infection, Aerobic bacteria, Antimicrobial susceptibility, Drug resistance

## Abstract

**Background:**

Post surgical wound infections are global problem in the field of surgery associated with long hospital stay, higher treatment expenditure, morbidity and mortality. Hence to address the limited data in Ethiopia on post surgical wound infections, we conducted this research to determine the prevalence and antimicrobial susceptibility patterns of aerobic bacteria in post-surgical wound infected patients in Ayder teaching and referral hospital, Mekelle, Ethiopia.

**Methods:**

Hospital based prospective cross sectional study was carried-out in 128 patients who had undergone surgery in general surgery and orthopaedic wards, and showed symptoms of infection clinically from January to June 2012. Standard bacteriological methods were used for bacterial isolation and antimicrobial susceptibility pattern.

**Results:**

A total of 128 patients (98 male and 30 female) with clinical signs of post surgical wound infections were enrolled. The age of the patients ranged from 15–79 years (with mean 35.95 ± 19.01 years). Out of the 128 wound swabs taken, 96/128 (75%) were culture positive aerobically, yielding 123 bacterial isolates. Out of these the predominant bacterial isolates were *Staphylococcus aureus* 44 (35.77%), Klebsiella species 29 (22.76%) and Coagulase negative *Staphylococci* (CoNS) 18 (14.63%). No bacterial isolates was found to be sensitive to all antibiotics tested. Isolated bacteria showed 102/123 (82.92%) multi drug resistance to the commonly used antibiotics in the hospital. However, 54/ 65 (83.1%) of Gram negative and 58/58 (100%) of Gram positive isolates were sensitive to Gentamicin and Vancomycin, respectively.

**Conclusion:**

Prevalence of was Post-operative wound infections rate in this current study was 75% and multi drug resistance was seen in 102/123(82.92%) of the isolates leaving clinicians with few choices of drugs for the treatment of post surgical wound infected patients. This underscores for periodic surveillance of etiologic agent and antibiotic susceptibility to prevent further emergence and spread of resistant bacteria pathogens.

## Background

Post-operative wound infections are major global problem in the field of surgery leading to many complications, increased morbidity and mortality [1, 2]. Most post-surgical wound infections are hospital acquired and vary from one hospital to the other [3]. Lack of standardized criteria for diagnosis presents a challenge to monitor the global epidemiology of surgical site infection. In addition to this, emerging of high anti-microbial resistance among bacterial pathogens has made the management and treatment of post-operative wound infections difficult [4]. The situation is serious in developing countries due to irrational prescriptions of antimicrobial agents [5]. The inoculum size, virulence and invasive capability of the organisms have been reported to influence the risk of infection. Moreover, the physiological state of the tissue in the wound and immunological integrity of the host also has equal importance in determining occurrence of infection [6, 7].

In Ethiopia, different studies reported that the prevalence of post surgical wound infection ranges from 14.8% -60% [8–12]. *S. aureus*, *Kelbsiella* species, *E. coli*, *Proteus* species, *Streptococcus* species, E*nterobacter* species, *Pseudomonas* species and Coagulase negative *Staphylococci* were reported as the most common pathogens [10, 11].

*P.aeruginosa* is an epitome of opportunistic nosocomial pathogen, which causes a wide spectrum of infections and leads to substantial morbidity in immuno compromised patients. Due to its high drug resistance to many antibiotics, the mortality rate is substantial [12, 13]. It is known that specific therapeutic options to patients with post surgical wound infections are mainly depend on data from antimicrobial susceptibility tests generated by clinical laboratories or sound epidemiological data from ongoing nosocomial infection surveillance [11]. However, data on the spectrum of bacteria isolated from hospitalized patients and their antimicrobial susceptibility patterns to guide post operative wound infection in the region is scarce. Furthermore, the magnitude and impact of multidrug resistance bacteria from post wound infections are unknown in Ayder referral and teaching hospital. Thus, this study aimed at determining the prevalence and drug susceptibility pattern of aerobic bacterial pathogens in post surgical wound infection in Ayder referral and teaching hospital.

## Methods

### Study design and sampling process

Hospital based cross sectional study was conducted in Ayder referral and teaching hospital from January to June 2012. The hospital is the only referral in the region serving for about 9 million people in including the neighbouring regions.

The study population consisted of patients who undergone surgery and developed SSI as diagnosed clinically by physicians within 30 days of having post surgical procedure. The sample size was estimated considering 92% prevalence (20), 5% precision, and 95% confidence level; with 10% contingency, thus, a total of 128 patients were included. During the study period, a total of 610 patients had undergone surgery, out of which swab samples were collected from 128 who showed clinical sings of post surgical wound infection.

Whereas surgical patients with community-acquired pyogenic infections such as abscess, furuncle and carbuncles; patients with infection of an episiotomy; and patients with open fractures were not included the study.

Postoperative wound infection: is defined as an infection in the tissues of the incision and operative area that can commonly occurs between the fifth and 30th days after surgery.

### Data collection and laboratory procedures

Demographic and clinical characteristics from patients were collected using structured questionnaire. Infected site was cleaned using normal saline and sterile gauze then, two wound swabs were collected using sterile cotton swabs from each patient and immediately transported to the laboratory.

### Bacterial identification

Wound swabs were processed in the bacteriology laboratory of the department of Medical Microbiology, Mekelle University, College of Health Sciences, within 1 hour of collection.

The first wound swab was used to make Gram stain smears whereas the second one was inoculated into blood agar, MacConkey agar and mannitol-salt agar, and incubated at 37°C for 24–48 hours. Identification of Gram positive bacteria was done using Gram stain, hemolytic activity on sheep blood agar plates, catalase reaction and coagulase test for Gram-positive bacteria. Gram-negative bacteria were identified based on colony morphology on blood agar and MacConkey agar, followed by biochemical reactions namely oxidase, triple sugar iron (TSI), Sulphur Indole and motility (SIM), citrate and urease tests [14].

### Drug susceptibility tests

Following identification of the bacterial isolates, a standard disc diffusion technique for drug susceptibility test (DST) was performed as recommended by Clinical and Laboratory Standard Institute (CLSI) [15]. Ampicillin (10 μg), Gentamicin (10 μg), Erythromycin (15 μg), Amoxicillin- clavunilic acid (30 μg), Ceftriazone (30 μg) Cloxacilline (5 μg) (Cypress) and Vancomycin (30 μg) were used for Gram positive isolates. Ampicillin (10 μg), Gentamicin (10 μg), Amoxacillin-clavunilic acid (30 μg), Amoxicillin (30 μg), Tetracycline (30 μg) Ciprofloxacin (5 μg) and Ceftriazone (30 μg) (all Oxoid, England) Gram negative isolates were tested.

### Quality control

Reference strains *P.aeruginosa* (ATCC-27853), *E.coli* (ATCC-25922) and *S.aureus (ATCC-*25923) were used as reference strains for identification and drug susceptibility testing.

### Data analysis

SPSS version 16 soft ware was used for statistical analysis. Chi-square test was used to determine the relationship between the dependent and the independent variables. P value <0.05 was considered as statistically significant.

### Ethical issues

The study was approved and ethically cleared by the Research and Ethical Review Committee of Mekelle University, College of Health Sciences (# REC REF 2012–127).

Written informed consent was obtained from each study participants and parents or care takers. All patient information was kept confidential and secret using codes.

## Results

We followed 610 successive patients (467 males and 143 female) who were referred for surgery to the hospital during the study period. From these, 128 patients were included (98 male and 30 female) with clinical sings of post surgical wound infections. The age of the patients ranged from 15–79 years (mean 35.95 ± 19.01 years). Bacterial growth was seen in 96/128(75%) of the patients yielding a total of 123 bacterial isolates. Among the isolates, 58(47.2%) were Gram positive and 65 (52.8%) were Gram negative. *S. aureus* 44 (35.77%) and *Klebsiella* species 29(22.76%) were the dominant isolates (Table 1). Single bacterial isolates were recovered from 73/96(76.05%) patients whereas 23/96(23.95%) had polymicrobial infections. *S. aureus* and *Proteus* species was the most common association (7 cases). Other frequent associations were: *S. aureus* and *Klebsiella* species (6 cases), *Proteus* species and *CoNS* (5 cases), *S. aureus* and *CoNS* (2 cases*), CoNS* and *E.coli* (2 cases) and *Klebsiella* species *and CoNS* (1 case). Sixty (46.87%), 36(28.12%) and 31(24.2%) of the patients were given Ceftriazone, Cloxacilline and combination of both, respectively for pre-surgical prophylaxis.

**Table 1:** **Frequency of aerobic bacteria from post-operative wound infection at Ayder Teaching and Referral Hospital, Mekelle, Ethiopia (January to June 2012)**

No.	Microorganism	Frequency N (%)
1	*S. aureus*	40 (34.2)
2	*Klebsiella* spp.	29 (24.8)
3	CoNS^a^	18 (15.4)
4	*Proteus spp.*	15 (12.8)
5	*P. aeruginosa*	11 (9.4)
6	*E. coli*	6 (5.1)
7	*Citrobacter* spp.	4 (3.4)
	Total	123 (100)

^a^Coagulase negative staphylococci.

Drug resistance of isolated Gram negative bacteria, irrespective of species/genus, was 92.3% to Ampicillin, 92.3% to Tetracycline and 92.3% to Amoxicillin, 81.5% to Ceftriazone, 69.2% to Amoxicillin Clavunilic acid, 46.2% for Ciprofloxacin, 26.2% to Erythromycin and 16.9% for Gentamicin. *Klebseilla* species showed 100%, 93.1%, 89.7% and 86.2% resistance for Amoxicillin, Tetracycline and Ceftriazone, respectively. *P. aeruginosa* isolates were 100% resistant for Ceftriazone, Amoxicillin, Amoxicillin clavunilic acid and Tetracycline. All *P. aeruginosa* isolates were; however, 100% sensitive to Gentamicin. All the 15 (100%) *Proteus*species were resistant for Amoxicillin and Tetracycline whereas, Gentamicin was 12/15 (80%) sensitive. Isolated *E.coli* showed 100% resistance to Amoxicillin -clavulunic acid, Tetracycline and Ampicillin, whereas, all of them 6 (100%) were sensitive for Gentamicin.

Isolated *Citrobacter* species were 100% sensitive to Gentamicin, while all 4(100%) of them were resistant to Ampicillin (Table 2).

**Table 2:** **Antimicrobial resistance pattern of Gram-negative aerobic bacteria in SSI patients attending Ayder Referral Hospital, Mekelle, Ethiopia (January to June 2012) No (%)**

Antibiotic	***Klebsiella spp.***	***P. aeruginosa***	***Proteus***spp.	***E.coli***	***Citrobacter***spp.	Total
	(n = 29)	(n = 11)	(n = 15)	(n = 6)	(n = 4)	(n = 65)
Ceftriazone	25 (86.2)	11 (100)	11 (73.3)	4 (66.7)	2 (50)	53 (81.48)
Erythromycin	13 (44.8)	3 (36.4)	9 (60)	2 (33.3)	2 (50)	17 (26.2)
Amoxicillin	29 (100)	11 (100)	15 (100)	4 (66.7)	3 (75)	62 (92.3)
Amoxicillin	19 (65.5)	11 (100)	7 (46.7)	6 (100)	2 (50)	45 (69.2)
clavunilic acid						
Gentamicin	8 (27.8)	------------	3 (20)	-------	------	11 (16.9)
Tetracycline	27 (93.1)	11 (100)	13 (86.7)	6 (100)	3 (75)	60 (92.3)
Ampicillin	26 (89.7)	11 (100)	13 (86.7)	6 (100)	4 (100)	60 (92.3)
Ciprofloxacin	11 (37.9)	9 (81.8)	7 (46.7)	1 (16.7)	2 (50)	30 (46.2)

Resistance by *S.aureus* was 36/40 (90%) to Tetracycline, Ceftriazone and Ampicillin, and 34/40 (85%) to Cloxacilline. All of the isolates *S.aureus* 40(100%) were sensitive for Vancomycin. High resistance rate of CoNS was observed for Amoxicillin, Amoxicillin-clavunilic acid, Ampicillin and Tetracycline, 88.9%, 77.8%, 77.8% and 77.8%, respectively.

All isolates of CoNS 18(100%) were however, sensitive for Vancomycin (Table 3).

**Table 3:** **Antimicrobial resistance pattern of Gram-positive aerobic bacteria in SSI patients at Ayder Referral Hospital, Mekelle, Ethiopia (January to June 2012) No (%)**

Antibiotic	***S.aureus***(n = 40)	CoNS (n = 18)	Total (n = 58)
Cloxacilline	34 (85)	14 (77.8)	48 (82.8)
Erythromycin	9 (22.5)	9 (50)	18 (30)
Amoxicillin	37 (82.5)	16 (88.9)	49 (84.5)
Amoxicillin clavunilic acid	20 (50)	14 (77.8)	34 (59.6)
Gentamicin	4 (22.2)	9 (50)	16 (27.6)
Tetracycline	36 (90)	14 (77.8)	50 (86.2)
Ceftriazone	36 (90)	13 (72.3)	41 (71.7)
Ampicillin	36 (90)	14 (77.8)	40 (70)
Vancomycin	-----	----	----

CoNS = Coagulase negative staphylococci.

In the univariate analysis, statistically significant association was not seen between sex and bacterial infection though more bacteria were recovered from male (P = 0.055). Age of the patient has no significant association with the frequency of bacteria isolation (*P* =0.064).

On multivariate logistic regression analysis, longer duration from admission to discharge, longer duration of pre-operative, wound type, ward type and type of operation were statistically associated with bacteria isolation from post operative wound infection (Table 4).

**Table 4:** **Multivariate logistic regression analysis for factors associated with bacterial isolation from post operative patients at Ayder Referral Hospital, Mekelle, Ethiopia (January to June 2012)**

Variable		Bacteria isolated from SSI (n = 96)	AOR [95% CI]	P value
Age (years)	15-30	19 (19.8)	2.07 (0.73-2.64)	
	31 – 40	25 (26.04)	3.83 (0 .42- 1.61)	0.064
	41-50	22 (22.9)	1.03 ( 0.24- 2.87)	
	≥ 50	30 (31.3)	2.84 ( 0.86- 2.67)	
Sex	Male	54 (56.3)	1.8 (0.96-4.19)	
	Female	42 (43.7)	1.42 (0 .21- 0.82)	0.055
Wound type	Class I	9 (9.4)	2.08 (0.92-1.46)	
	Class II	17 (17.7)	2.43 (0.72-3.46)	0.029
	Class III	29 (30.2)	3.92 (1.73 - 5.77)	
	Class IV	41 (42.7)	5.41 (2.81-4.91)	
Ward type	General Surgery	26 (27.1)	2.78 ( 0.93-1.25)	
	Orthopaedic	70 (72.9)	4.13 (1.18 – 3.10)	0.012
Operation type	Elective	35 (36.5)	1.95 (1.24 - 4.65)	0.038
	Emergency	61 (63.5)	5.71 (2.09-5.38)	
Duration from	≤ 1 day	39 (40.6)	0.83 (0.72-3.46)	0.014
admission to operation	> 1 days	57 (59.4)	3.83 (1.94 - 2.07)	
Duration from operation	<14 days	33 (34.4)	2.06 (0.72-3.46)	0.008
to discharge	>14 days	63 (65.6)	4.92 (3.62-6.96)	
Pre-operative	Cloxacilline	60 (46.87)	1.51 (1.92-3.06)	
chemoprophylaxis	Ceftriazone	36 (28.1)	3.91 (2.67-4.01)	0.021
	Cloxacilline+	31 (24.2)	3.16 (2.62-4.46)	
	Ceftriazone			

## Discussion

Wound infections rate in this present study was 75% which corroborates the reports from Niger 74.9% [16] and Nepal 80% [2] and rural tertiary hospital in Nigeria 70.1% [3] but higher than Addis Ababa 14.8% [8], North Ethiopia 44.1% [12], Kenya 7% [17] and tertiary care hospital in Gujarat, India 68.85% [13]. However, our finding was lower than reports from India 84% [18] , South Ethiopia 92% [19] and West Ethiopia 96.3%( 10). This difference in prevalence of post-operative infection may be due to variation in common nosocomial pathogens inhabitant, difference in policy of infection control and prevention between countries and hospitals, and study design used in the researches.

Multiple infections among post surgical wound infected patients was seen in 23(23.95%) of patients where S*. aureus* and *Proteus* species was the most common association where are the rest 77(87.5%) have shown single bacterial infection. *S.aureus* followed by *Klebsiella* spp was the predominant isolate which is in agreement to findings from Kenya [17] and Ethiopia [19]. *S. aureus* was the most prevalent isolates. This was in line with previous surveillance conducted in Ethiopia [10], Uganda [1], India [18] and Niger [20]. The normal flora nature of *S.aureus* in the skin and anterior nares, which can enter to deep site during surgery of the natural barrier of the skin, could be the possible justification for its high prevalence [1, 21].

Again the high prevalence rate of Entero-bacterial isolates in the current study could reveal faecal contamination due to poor personnel hygiene [16] or due to post procedural contamination [14].

Bacterial growth was not seen in 32/128 (25%) patients, which could attribute to the normal healing process of the wound by host immune system, antimicrobial activity or appropriate use of antiseptics for cleaning the wounds. It could also be due to anaerobic bacteria or fungi infection which we could miss due to the use of culture media that only support the aerobic bacteria [22, 23].

Patients who were given pre-operative antibiotic prophylaxis (Cloxacilline, Ceftriazone or both) developed more surgical site infections and drug resistance for these drugs. This was similar with the study done in Ethiopia by Taye (8) where patients who received preoperative antibiotics have a statistically significant higher wound infection rate (p < 0.001).

The present in vitro antimicrobial sensitivity test showed that isolated bacteria react differently to various antibiotics. Thirty six (90%) of the isolated *S.aureus* in our study were sensitive to Gentamicin similar to the report from Ethiopia [19, 24], Nepal [2] and Uganda [1]. However, 80% resistance of *S.aureus* to Gentamicin was reported elsewhere [25].

The difference could be due to the difference in prescribing this antibiotic for the treatment of the bacteria from hospital to hospital. All the isolated *S.aureus* 40(100%) were susceptible to Vancomycin in our finding exactly the same with that of report from Nepal 100% [2] and from West Ethiopia 100% [24]. But resistance of 66.7% to Vancomycin was seen elsewhere [12, 19]. Higher resistance 90% of *S.aureus* for Ceftriazone was observed in our current study than others [2, 19, 26]. High resistance rate (98.6-100%) was seen by *Klebsiella* spp. to Ceftriazone, Ampicillin and Amoxicillin similar to the report by from Ethiopia [24]. Less resistance 27.8.2% was observed to Gentamicin similar to study done elsewhere [2], but in contrary to our result, 100% resistance was reported from India [26]. The isolated *P.aeruginosa* were 100% resistant to Ceftriaxon, Amoxicillin, Tetracycline and Ampicillin similar resistance was reported elsewhere [19, 26] but as reported from Nepal [2] all isolates were 100% susceptible for Gentamicin.

Isolated *E. coli* were 100% resistant to Ampicillin similar result for Ampicillin was seen from Uganda [1], but all of them (100%) were sensitive for Gentamicin, similar to the result elsewhere [1, 19, 24]. *Citrobacter* spp were 100% resistant to Ampicillin exactly similar to the findings from Ethiopia 100% [24]. They were; however, all 100% sensitive to Gentamicin which is opposite to the result from Ethiopia [24] where all isolates were 100% resistant. Possible explanations for the disparity in drug resistance over places could attribute to the difference in the rational use of antibiotics in the study areas. In general multiple antibiotics resistance was seen in 81.4% of Gram negative and 82.8% of the Gram positive isolates. All isolated bacteria were highly resistant to Ampicillin, Ceftriazone, Tetracycline, Amoxicillin, Amoxicillin- clavunic acid and Cloxacilline. This remarkably higher resistance may be due to their easily availability and indiscriminate use of the drugs without proper prescription. Vancomycin, Gentamicin and Erythromycin in our study appear to be effective against post surgical wound infection in the study area that can be used with caution. On multivariate logistic regression analysis, longer duration from admission to discharge, longer duration of pre-operative, wound type, ward type and type of operation were statistically associated with bacteria isolation from postsurgical wound infection which in lines with studies done elsewhere [27, 28]. This is because patients with wound class III/contaminated (open, fresh, accidental wounds) and class IV/dirty-Infected (old traumatic wounds with retained devitalized tissue) and those that involve existing clinical infection or perforated viscera are at high risk to develop SSI (30). Orthopaedic wards, since patients require longer hospitalization time to recover from their bone cases, they become prone for post operative wound infections. In our present study patients who underwent emergency surgery type operation were at higher risk that those who underwent elective type operation. This may probably, due to the lack of readiness for operation on the patients’ side (8).

### Limitation

The study did not isolate strict anaerobes bacteria and fungi, which could have increased the number of bacterial isolates reported as negative cultures.

## Conclusion

Surgical site wound infection was high (75%) and multi drug resistance was seen in 102/123 (82.92%) of the isolates leaving clinicians with few choices of drugs for the treatment of post surgical wound infected patients. Wound type, longer preoperative stay, type of operation, wound class and ward type, showed statistically significant association with postoperative wound infection. Therefore, periodic surveillance of bacteria and antibiotic susceptibility is important to prevent further emergence and spread of resistant bacteria pathogens.

## Authors’ information

Araya Gebreyesus Wasihun (BSc in medical lab Sciences, MSc in medical micro biology) Dr. Reiye Esayasis Mengesha (MD^+^, Ass. Professor) a senior general surgeon.

Dr. Berhe Gebre-Slassie Kasa (MD^+^, Ass. professor) is the only orthopedic surgeon in the hospital and department head of orthopedic surgery.

Dr Muthupandian Saravanan (PhD, Ass. Professor in medical microbiology and currently in the department of medical microbiology and immunology).

Mr. Derbew Fikadu Berhe (MSc, Ass.Professor in pharmacy, and PhD fellow in Netherlands.
